# The outcomes of controlled ovarian hyperstimulation/intrauterine insemination in patients with unilateral tubal occlusion on hysterosalpingograph

**DOI:** 10.4274/tjod.88786

**Published:** 2016-03-10

**Authors:** Selçuk Selçuk, Mehmet Küçükbaş, lter Yenidede, Semra Kayataş Eser, Ahmet Eser, Çetin Çam, Hüseyin Tayfun Kutlu

**Affiliations:** 1 Zeynep Kamil Women and Children’s Diseases Education and Research Hospital, Clinics of Obstetrics and Gynecology, İstanbul, Turkey; 2 Fatih Sultan Mehmet Training and Research Hospital, Clinic of Obstetrics and Gynecology, İstanbul, Turkey

**Keywords:** Unilateral tubal occlusion, infertility, intrauterine insemination

## Abstract

**Objective:**

The aim of the present study was to evaluate the pregnancy rates of intrauterine insemination (IUI) and controlled ovarian hyperstimulation (COH) in patients with one-sided tubal occlusion on hysterosalpingography (HSG).

**Materials and Methods:**

Patients who underwent COH/IUI were enrolled into this retrospective cohort study. The patients with one-sided tubal occlusion diagnosed under HSG who met the inclusion criteria were accepted into the study group. The control group consisted of patients with unexplained infertility. The outcomes of COH/IUI were compared between the study and control groups.

**Results:**

Ninety-seven patients in the study group (n=44) and control group (n=53) who underwent COH/IUI treatment were included into study. The biochemical, clinical, and ongoing pregnancy rates were similar between patients with unilateral occlusion diagnosed under HSG and those with unexplained infertility. The spontaneous pregnancy rate within one year was higher in patients with normal HSG than in patients with unilateral tubal occlusion, but the difference did not show statistical significance.

**Conclusion:**

Infertile patients with one-sided tubal occlusion in HSG can be managed as with patients with unexplained infertility and normal HSG findings. In addition, COH/IUI may be considered as the first-line treatment option in the management of these patients.

## INTRODUCTION

Tubal disease is responsible for approximately 30-40% of female infertility^(1)^. Recently, the incidence of tubal factor has increased and has become a major cause of female infertility. The major risk factor for tubal factor infertility is pelvic inflammatory disease; other possible risk factors are history of tubal surgery and ectopic pregnancy^(2)^. Hysterosalpingography (HSG) and laparoscopy are the most common procedures used in the assessment of the tubal patency. HSG is usually the first preferred clinical tool because laparoscopy is more invasive and more expensive^(3)^. There is no consensus about the optimal management of patients with unilateral tubal occlusion. The assessment of tubal patency through laparoscopic chromotubation, intrauterine insemination with controlled ovarian stimulation (COH), and in vitro fertilization (IVF) are the recommended management options for these patients^(4,5,6)^. In the literature, there are insufficient data regarding the success rates of COH and IUI in the treatment of patients with unilateral tubal occlusion.

In the present study, we aimed to evaluate the pregnancy rates of COH/IUI in patients with unilateral tubal occlusion diagnosed under HSG.

## MATERIALS AND METHODS

This retrospective cohort study was conducted in Zeynep Kamil Tertiary Hospital between 2013 and 2015. The study protocol was approved by the Local Research and Ethics Committee of the institution. Demographic and clinical information of patients were abstracted from the hospital’s database. Inclusion criteria were age ≥18 and <40 years, basal follicle-stimulating hormone (FSH) level <15 IU/mL, normal basal luteinizing hormone, body mass index <35 kg/m^2^, normal semen parameters according to the World Health Organization (WHO) criteria, no presence of endocrine abnormalities, and no uterine cavity abnormalities^(7)^. Patients with unilateral tubal occlusion diagnosed under HSG and who met the inclusion criteria were accepted into the study group. The control group consisted of patients with unexplained infertility, normal HSG findings and those who met the same inclusion criteria. All patients underwent COH/IUI treatment.

In the ovarian stimulation protocol, subcutaneous injection of gonadotropins as recombinant FSH (Gonal F, Merck Serono, İstanbul, Turkey) with starting dose of 50-100 IU/day from the 2^nd^-4^th^ day of the menstrual cycle was administered. Monitoring using transvaginal ultrasonography (TVU) was performed daily after the fifth day of stimulus. When ≥2 follicles reached a diameter of ≥17 mm, subcutaneous injection of recombinant chorionic gonadotropin alpha 250 mg (Ovitrelle; Merck-Serono, İstanbul, Turkey) was administered. A concentrated, washed sperm sample was prepared and IUI was performed 34-36 hours after human chorionic gonadotropin (hCG) injection.

Primary outcomes were biochemical, clinical, and ongoing pregnancy rates. The secondary outcome was the spontaneous pregnancy rate. Patients were invited to the infertility clinic to measure the β-hCG value 15 days after IUI. Positive serum β-hCG levels as ≥10 mIU/L were regarded as biochemical pregnancy and presence of a gestational sac on ultrasonography was regarded as clinical pregnancy. Ongoing pregnancy was defined as a pregnancy ≥12 weeks of gestation. Spontaneous pregnancy was accepted as pregnancy without any treatment within one year after unsuccessful IUI.

### Statistical Analysis

Statistical analysis was performed using SPSS version 15.0 software. Mann-Whitney U test and Pearson’s Chi-square tests were performed where appropriate. A value of p=0.05 was accepted as the degree of significance. Data are given as mean ± standard deviation or percentage.

## RESULTS

Ninety-seven patients who met the inclusion criteria and underwent COH/IUI treatment were included in study. Forty-four patients with unilateral tubal occlusion were included into the study group and 53 patients with unexplained infertility were assigned as the control group. Comparison of baseline clinical characteristics and sperm parameters of the two groups are given in Table 1. There were no significant differences between the study and control groups. In addition, there were no significant differences regarding IUI cycle characteristics of patients when the two groups were compared (Table 1). The biochemical, clinical, and ongoing pregnancy rates of the two groups are given in Table 2. The biochemical, clinical, and ongoing pregnancy rates per cycle of study group were 13.6%, 11.4%, and 11.4%, respectively. The biochemical, clinical, and ongoing pregnancy rates of the control group were 9.4% for all parameters. There were no statistical differences between the two groups. The spontaneous pregnancy rates were found 15.9% and 18.9% for study group and control group, respectively, and there was no statistically significant difference.

## DISCUSSION

In present study, the biochemical, clinical, and ongoing pregnancy rates of COH/IUI treatment were similar between patients with unilateral occlusion and patients with unexplained infertility. In addition, the spontaneous pregnancy rate within one year after unsuccessful IUI treatment was higher in the control group than in the study group but the difference did not reach statistical significance.

The management of infertile patients showed differences based on their HSG findings. In general, patients with bilateral tubal occlusion can be referred to IVF treatment or for further evaluation for tubal patency with laparoscopic chromotubation. The management of patients with one-sided tubal occlusion is less clear^(1,8)^.

In the literature, the diagnostic accuracy of HSG was evaluated in various studies. Mol et al.^(9)^ conducted a prospective cohort study of 794 patients with the participation of 11 clinics to evaluate the importance of HSG and laparoscopy for the prediction of fertility outcomes. The sensitivity and specificity of HSG was reported as 0.81 and 0.75, respectively, for any form of tubal occlusion at laparoscopic surgery.

The authors reported the adjusted fecundity rate ratios (FRR) for unilateral tubal occlusion diagnosed at HSG as 0.80 and for bilateral tubal occlusion as 0.49. Accordingly, the authors concluded that bilateral tubal occlusion significantly impaired fertility outcomes whereas unilateral tubal occlusion mildly reduced fertility outcomes. Diagnosis of occlusion at laparoscopy had a greater worsening effect on fertility outcomes (FRR=0.51 and 0.15 for unilateral and bilateral tubal occlusion, respectively) than those at HSG^(9)^.

A retrospective study assessed the fertility prognosis of patients with tubal occlusion detected using HSG. The FRR of unilateral tubal occlusion was 0.81 and that of bilateral tubal occlusion was 0.30^(10)^. The authors suggested that patients with one-sided tubal pathology and patients with normal HSG findings had nearly similar fertility potential, but the presence of bilateral tubal pathology detected on HSG decreased fertility potential significantly. In our study, the spontaneous pregnancy rate of patients with normal HSG findings was higher than patients with one-sided tubal occlusion, but the difference was not found as statistically significant (18.9% vs. 15.9%, p>0.05).

In the literature, the success rates of COH/IUI in patients with diagnosis of unilateral tubal occlusion at HSG were assessed in different studies. In a retrospective study, Lin et al.^(2)^ reported that the pregnancy rates per cycle of COH/IUI treatment for patients with one-sided tubal occlusion on HSG and those with normal HSG findings were 17.3% and 18.9%, respectively. The difference of pregnancy rates between the two groups showed no statistical significance. The authors stated that COH/IUI could be initial treatment options for infertile patients with unilateral tubal occlusion. Farhi et al.^(11)^ assessed the cumulative pregnancy rates for three cycles of COH/IUI treatment among patients diagnosed as having one-sided tubal occlusion compared with patients with unexplained infertility (controls). The cumulative pregnancy rates were reported as 30.9% for the study group and 42.6% for the control group. The authors stated that there was no significant difference between the two groups in terms of cumulative pregnancy rates. Yi et al.^(12)^ evaluated the outcomes of COH/IUI treatment among thirty-seven infertile women (52 cycles) with unilateral tubal occlusion compared with a control group that included patients with unexplained infertility. The pregnancy rate per cycle was 17.3% in patients with unilateral tubal occlusion and 16.5% in the control group without statistical significance. The outcomes of our study are similar with the literature. In the present study, the ongoing pregnancy rate per cycle of study group was 11.4% whereas the ongoing pregnancy rate per cycle of control group was 9.4%. The biochemical, clinical, and ongoing pregnancy rates per cycles did not show a significant difference between the study and control groups.

Conflicting results about the clinical importance of the site of tubal occlusion and outcomes of COH/IUI treatment are reported in different studies. Lower pregnancy rates are demonstrated in women with mid-distal or distal tubal occlusion than in women with proximal tubal occlusion(^2,11,12)^. Some authors stated that the site of tubal occlusion should be considered in the management of patients with unilateral tubal occlusion whereas others reported that the site of tubal occlusion had no importance in the management of these patients^(2,11,12)^.

The relatively small sample size and retrospective nature of the study were the limitations of the present study.

## CONCLUSION

Infertile patients with one-sided tubal occlusion on HSG can be managed as with patients with unexplained infertility and normal findings on HSG. In addition, COH/IUI may be considered as the first-line treatment option in the management of these patients.

## Figures and Tables

**Table 1:**
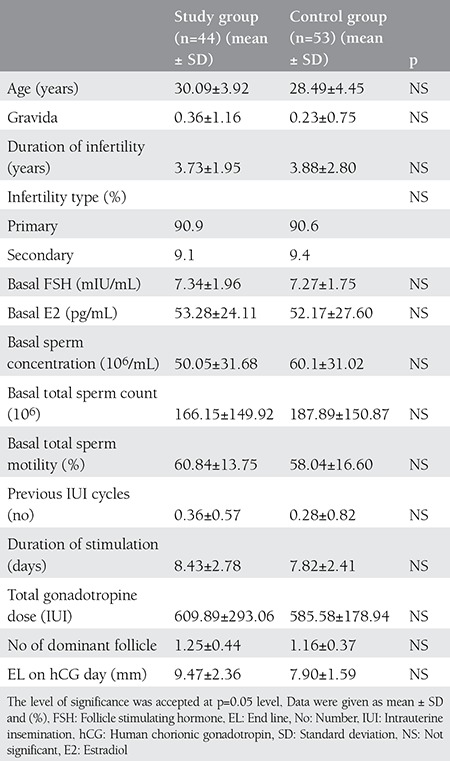
Comparison of demographic and clinical characteristics between the two groups

**Table 2:**
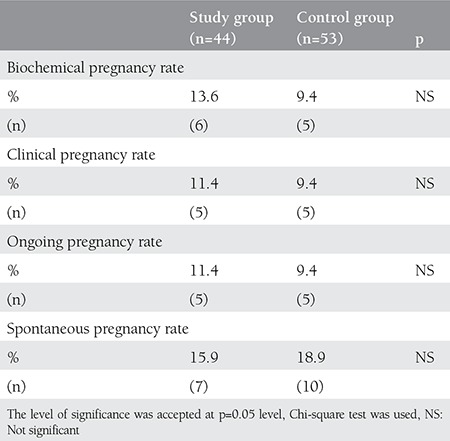
Comparison of outcomes of intrauterine insemination cycles between two groups
